# Short- and long-term stability of synovial fluid calprotectin

**DOI:** 10.11613/BM.2024.030704

**Published:** 2024-10-15

**Authors:** Helena Čičak, Stjepan Bulat, Joško Jeličić, Alan Ivković, Ksenija Maštrović Radončić, Vanja Radišić Biljak, Lora Dukić, Ana-Maria Šimundić

**Affiliations:** 1 Department of Medical Laboratory Diagnostics, University Hospital Sveti Duh, Zagreb, Croatia; 2 Department of Orthopaedic Surgery and Traumatology, University Hospital Sveti Duh, Zagreb, Croatia; 3 School of Medicine, University of Zagreb, Zagreb, Croatia; 4 Department of Clinical Medicine, School of Applied Health Sciences, University of Zagreb, Zagreb, Croatia; 5 Polyclinic Ivković, Zagreb, Croatia; 6 Department of Rheumatology, Physical and Rehabilitation Medicine, University Hospital Sveti Duh, Zagreb, Croatia; 7 Faculty of Kinesiology, University of Zagreb, Zagreb, Croatia; 8 Clinical Department of Laboratory Diagnostics, University Hospital Center Rijeka, Rijeka, Croatia; 9 Faculty of Pharmacy and Biochemistry, University of Zagreb, Zagreb, Croatia; 10 Department of Global Medical & Clinical Affairs, Business Unit for Preanalytics, Greiner Bio-One GmbH, Kremsmünster, Austria

**Keywords:** calprotectin, preanalytical phase, stability, synovial fluid

## Abstract

**Introduction:**

Information about analyte stability is of crucial importance. The aims of this study were to determine the short- and long-term stability of synovial fluid calprotectin at various temperature conditions (4-8 °C for 7 days, - 20 °C and - 80 °C for 6 weeks).

**Materials and methods:**

Eleven samples from patients were included in this study. The samples were promptly transported at room temperature (RT) to the laboratory immediately after arthrocentesis. Upon arrival, the samples were transferred into plastic tubes without additives and pretreated with hyaluronidase solution. After centrifugation at 1500xg for 10 minutes at RT, the baseline calprotectin concentrations were determined. Seven aliquots were stored in LoBind tubes (Eppendorf) at 4-8 °C and the calprotectin was measured every day. Six additional aliquots were stored at temperatures - 20 °C and - 80 °C and the concentration of calprotectin was measured weekly. Analysis was done using Buhlmann fCAL turbo reagent on analyzer Siemens Atellica Solution (Siemens Healthcare, Erlangen, Germany). Data were analyzed by Microsoft Excel and MedCalc statistical software. The percentage difference (PD%) was calculated. The maximum permissible difference (MPD) was 9.1% for PD%.

**Results:**

The PD% with the corresponding 95% confidence intervals were inside the predefined MPD. The instability equations and correlation coefficient for storage temperatures were PD% = 0.1644 x time (day), r = 0.06, P = 0.614 for 4-8°C, PD% = 0.5190 x time (week), r = - 0.22, P = 0.080 for - 20°C, and PD% = 0.1316 x time (week), r = 0.08, P = 0.545 for - 80°C.

**Conclusions:**

The calprotectin in the synovial fluid is stable when stored long-term for 6 weeks at - 20 °C or at - 80 °C or short-term (7 days) at 4-8 °C.

## Graphical abstract

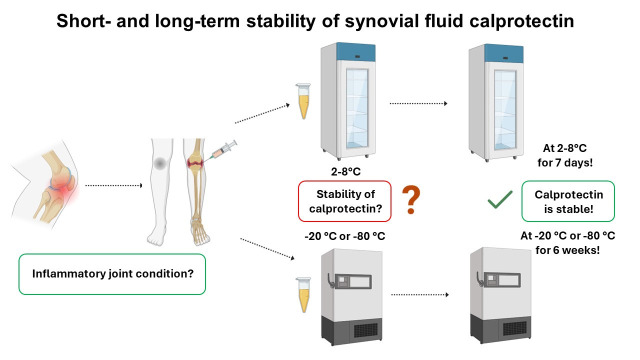

## Highlights

• Synovial calprotectin may have an important role in diagnosing inflammatory joint conditions

• This stability study was done according to the Checklist for Reporting Stability Studies (CRESS) recommendations

• The CRESS recommendations enabled better standardization among stability studies

• Calprotectin in synovial fluid samples is stable for 7 days at 4-8 °C

• Calprotectin in synovial fluid samples is stable for 6 weeks at - 20 °C and - 80 °C

## Introduction

Synovial fluid calprotectin may have an important role in diagnosing and differentiation between joint inflammatory and non-inflammatory conditions. Numerous joint diseases such as osteoarthritis (OA) or rheumatoid arthritis (RA) have an inflammatory background in their pathophysiology. Recent studies also indicated that calprotectin in the synovial fluid is a reliable marker for periprosthetic joint infection (PJI), with significant potential for use in clinical practice as a diagnostic test for PJI (*1*, *2*). Moreover, PJI as a complication after arthroplasty, is difficult to diagnose due to presence of several diagnostic guidelines and atypical symptoms, potentially leading to delays in proper treatment and decrease the odds of a good clinical prognosis (*1*). Most criteria for diagnosing joint diseases include the measurement of C-reactive protein (CRP) and erythrocyte sedimentation rate (ESR) which are inflammatory markers. However, these markers are non-specific and represent the systemic reaction. The new scoring-based definition for periprosthetic joint infection include an elevated synovial fluid leukocyte count, as well as elevated percentage of synovial polymorphonuclear leukocytes. Neutrophils constitute the majority of leukocytes in peripheral blood, and actively participate in the earliest phases of the inflammation process (*3*, *4*). Also, one of newest findings by Alkadhem MF *et al.*, was that the synovial calprotectin has higher accuracy than the use of leukocytes and polymorphonuclears in synovial fluid in excluding a chronic PJI which is one of great indication that synovial fluid could be use in routine clinic (*5*). Following neutrophil migration into the inflamed tissue, their function lasts only for 1-2 days before their degradation. Moreover, because of the cell fragility, the synovial fluid leukocyte count needs to be performed within one hour from the collection of the synovial fluid (*6*). As the neutrophil cytosol contains more than 50% of the calprotectin, it proves to be a highly useful biomarker for the presence of inflammatory cells within the specific tissues or body fluid (*e.g.,* synovial fluid) (*7*).

Several studies have aimed to investigate calprotectin concentration in synovial fluid in various joint diseases, *e.g.,* rheumatoid arthritis, osteoarthritis, periprosthetic joint infection *etc.* (*2*, *8*-*10*). Also, one study showed one of the first report of the use of serum calprotectin in distinguishing bacterial urinary infections and viral respiratory infections in children (*11*). This study gives a new light into the possible use of calprotectin in differentiating the conditions samples by bacterial and viral pathogens and other causes of the disease, including as well the use of calprotectin in synovial fluid in various joint infections. On the other hand, in these studies, calprotectin concentrations were measured using enzyme-linked immunosorbent assay (ELISA) method or by using point-of-care devices. In one study, concentration of calprotectin was measured by immunoturbidimetric assay (*12*). Although, most samples were frozen until analysis, the time between storage and analysis was not stated. Moreover, only two studies determined the stability of calprotectin in synovial fluid. A recent study on preanalytical considerations concerning synovial fluid calprotectin detected high stability of synovial fluid calprotectin during 7-day storage at + 4 °C and even at room temperature (RT) conditions (*13*). Other study determined stability of calprotectin only by analyzing two of the samples after three freeze/thaw cycles. This study showed that calprotectin was stable in those two samples, but the duration of storage is unclear (*7*).

On the other hand, there is no stability studies for calprotectin in synovial fluid that were done according to recent European Federation of Clinical Chemistry and Laboratory Medicine Working Group for the Preanalytical Phase (EFLM WG-PRE) Checklist for Reporting Stability Studies (CRESS) recommendations (*14*). The CRESS recommendations enabled higher level of standardization among biomarker stability studies. Determination of short- and long-term stability for calprotectin is an indispensable part of biomarker validation. Unavailable or unreliable data on biomarker stability significantly impact the reliability of research results, potentially leading to misleading study conclusions. Various factors, such as analyzer failure or the transport of the synovial fluid to distant laboratories may slow down sample processing. Information on stability in diverse storage conditions (*e.g*., different temperatures, durations of storage) is especially desirable, ensuring the applicability of stability information to most laboratories. For instance, knowledge of short-term stability can be useful for analyzing routine patient samples using automated analyzers or the ELISA method, whereas knowledge of long-term stability is preferred for biobanking or research purposes, which cannot or will not be performed in a short period of time.

The aims of this study were to determine short- and long-term stability of calprotectin, by CRESS recommendations, in following conditions: i) at temperature 4-8 °C for 7 days, ii) at - 20 °C for 6 weeks, and iii) at - 80 °C for 6 weeks.

## Materials and methods

**Figure 1:**
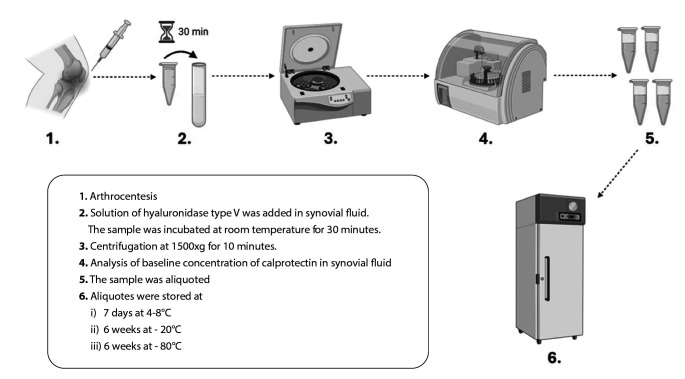
Flowchart of the procedure from sampling to storing aliquots in the stability study. The picture was created in BioRender.com.

The study was performed at the Department of Medical Laboratory Diagnostics of University hospital Sveti Duh (Zagreb, Croatia) and according to the EFLM WG-PRE CRESS checklist. Leftover samples from eleven individual patients with suspected inflammatory joint diseases who had a justified indication for arthrocentesis were included. The arthrocentesis was performed by an experienced orthopedic specialist. Synovial fluid samples, collected in syringes without additives, were instantly delivered to the laboratory at room temperature. The volume of the samples ranged was from 1.5 mL to 5 mL. Immediately upon receipt, the samples were aliquoted into plain plastic tubes (without additives) for the analysis of calprotectin concentration. After aliquoting, solution of hyaluronidase type V, lot SLCG7935 (Sigma Aldrich, Missouri, USA) was added in the sample according to the laboratory standard operating procedure for pretreatment of synovial fluid samples. The obtained results were not corrected with the dilution factor because it was 1.05, which is not clinically significant. The tubes were gently mixed 3-5 times and incubated for 30 minutes. The samples were centrifuged, and the baseline concentration of calprotectin was determined in the supernatant on Atellica Solution (Siemens Healthineers, Erlangen, Germany) analyzer. The Buhlmann fCAL turbo (Buhlmann Laboratories AG, Schonenbuch, Switzerland), a turbidimetric assay was used to measure the concentration of calprotectin in synovial fluid samples. The aforementioned assay has been validated and designed for stool samples, and it was used with official application settings as recommended by the reagent manufacturer for analyzer Atellica Solution for determination of fecal calprotectin (*15*). The only step which was changed was step after measurement, *i.e.* the division of already corrected results from analyzer with a dilution factor of 500. Although fecal samples need to be diluted in the ratio of 1:500 prior to analysis, there is no need for prior dilution of synovial fluid samples. In the application for fCAL turbo reagent for fecal calprotectin, after the measurement, the “original *i.e.* measured value” from analyzer was automatically multiplied with factor dilution 500 because of the extraction stool in 5 mL of buffer. By dividing the “final” result from analyzer, by a dilution factor of 500, the obtained result is the one who has been “originally” measured. Consequently, the result of synovial calprotectin obtained from the analyzer had to be divided by 500. After analyzing the baseline calprotectin concentration, aliquots of a minimal volume of 200 µL were stored in 1.5 mL Eppendorf tubes. As mentioned before, samples were stored at various time/temperature conditions: 7 days at 4-8 °C, 6 weeks at - 20 °C and as well as at - 80 °C. The new aliquot was taken for each time point and storage condition in this stability study. The flowchart of process from sampling to storing synovial samples is shown in Figure 1. The samples were stored in Infrico refrigerator model PTR50G (4-8 °C), Infrico freezer model LTF70S at - 20 °C (Infrico medcare, Lucena, Spain), and Cryocube UTF model F57H (at - 80 °C) (Eppendorf, Hamburg, Germany) all with a built-in system for temperature monitoring. Additionally, the temperatures were also verified by the laboratory personnel, in 12 hours shifts. The mean (minimum to maximum) temperature for all three storage temperatures during the storage periods were 4.7 °C (4.0 to 5.7 °C), - 18.1 °C (- 20.0 to - 13.4 °C), and - 79.7 °C (- 81.0 to - 79.0 °C). After the baseline concentration of calprotectin has been determined, seven aliquots were stored at 4-8 °C. Calprotectin concentration was measured consecutively every day for 7 days. Six aliquots were stored at temperatures - 20 °C and - 80 °C and the concentration of calprotectin was measured weekly, for 6 weeks. Prior to measurement, the aliquots were thawed and homogenised. All samples were analysed in duplicate.

Maximum permissible difference (MPD) value was used from reagent manufacturer declaration (Buhlmann fCAL turbo, B-KCAL-RSET, version A2) for the within-laboratory precision coefficient of variation (CV%) of 9.1% because of the lack of any other available criteria (*e.g.*, biological variation for calprotectin in synovial fluid) (*16*).

Furthermore, prior beginning this stability study, the analytical performance of precision on one synovial fluid sample was determined by Clinical and Laboratory Standards Institute (CLSI) EP15-A3 guidelines, and CV% for concentration of calprotectin 1.62 mg/L was 0.84% (unpublished data of the author) (*17*). Research involving human subjects complied with all relevant national regulations, institutional policies and is in accordance with the tenets of the Helsinki Declaration (as revised in 2013) and has been approved by the Ethical Board of the Sveti Duh University Hospital. (Registry number: 01-03-2589/6)

### Statistical analysis

The percentage difference (PD%) was calculated by this equation PD% = ((t_x_ - t_0_) / t_0_) x 100 where t_x_ represents the average concentration for certain time point and t_0_ was an average concentration in the baseline time point. For every time/temperature storage condition, the instability equations were obtained by regression analysis. The instability equation was expressed in form PD% = a x time, where a is the slope of the equation. For the detection of outliers, the CV% from all replicates was compared to the obtained CV% of interquartile range (IQR). If the CV% of replicates were three times higher than the obtained CV% of internal quality control (IQC), then it is considered as an outlier (*18*). Descriptive statistics and regression analysis was performed using MedCalc 20.027 (MedCalc software, Ostend, Belgium) software and data visualization was done using Microsoft Excel for Microsoft 365 MSO (version 2209).

## Results

**Figure 2:**
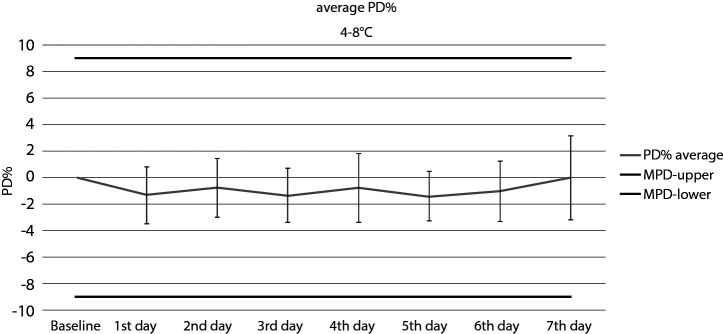
Point to point estimation with 95% confidence intervals for the average percentage difference (PD%) of all patients for every day for determing stability of calprotectin in synovial fluid when it stored at 4-8 °C for 7 days. The black lines represents maximum permissible difference (MPD).

**Figure 3:**
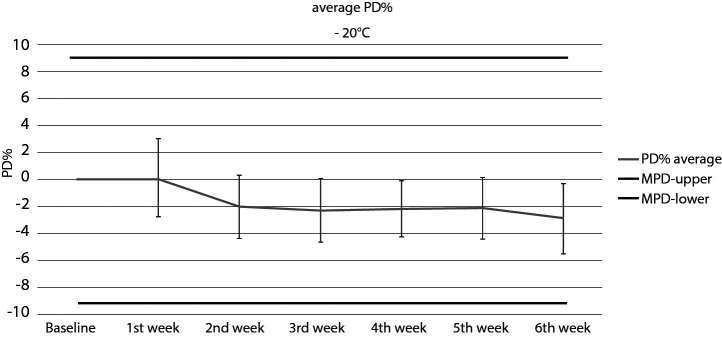
Point to point estimation with 95% confidence intervals for the average percentage difference (PD%) of all patients for every week for determing stability of calprotectin in synovial fluid when it stored at - 20 °C for 6 weeks. The black lines represents maximum permissible difference (MPD).

**Figure 4:**
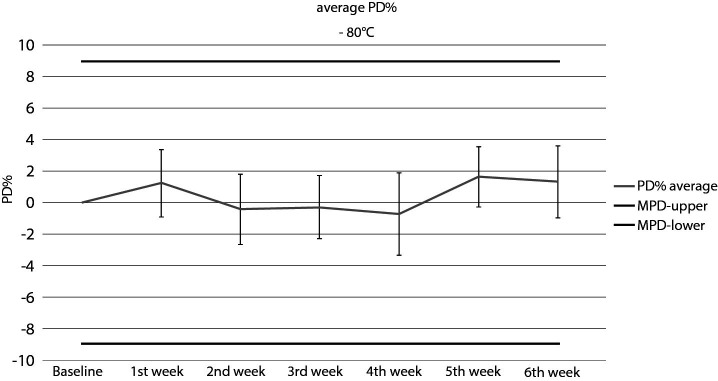
Point to point estimation with 95% confidence intervals for the average percentage difference (PD%) of all patients for every week for determing stability of calprotectin in synovial fluid when it stored at - 80 °C for 6 weeks. The black lines represents maximum permissible difference (MPD).

In this stability study, the median of baseline of calprotectin concentration was 1.48 mg/L with the IQR of 1.27 to 1.63 mg/L, for all 11 included samples. The mean values of replicates were used in further data analysis. The median CV% for all replicates of the samples for all temperatures was 0.51% with minimum and maximum values from 0 to 6.88%. No outliers were identified in the obtained results. The stated instability equations and PD% for each of the three storage temperatures are presented in Table 1, along with the 95% confidence intervals (95% Cl). According to the slope in the instability equations, the concentration of synovial fluid calprotectin showed decreasing trend for the samples stored at 4-8 °C and at - 20 °C. On the contrary, an increasing trend was observed for samples stored at - 80 °C. Additionally, graphical representations of the instability equations with 95% CI and point-to-point estimation with confidence intervals are provided for each time point at all three temperatures in and Figures 2-4, respectively. All the 95% CIs in the graphical representations are inside the upper and lower MPDs, ensuring that 95% of the samples will be stable over all three storage temperatures.

**Table 1:** Average PD% with 95% Cl and the instability equations for all storage conditions

**Storage temperature**	**Time of storage**							**Instability equations**
**4-8 °C**	**1st day**	**2nd day**	**3rd day**	**4th day**	**5th day**	**6th day**	**7th day**	
PD%	- 1.31 (- 3.47 to 0.85)	- 0.75 (- 2.97 to 1.48)	- 1.32 (- 3.32 to 0.68)	- 0.77 (- 3.36 to 1.83)	- 1.41 (- 3.29 to 0.46)	- 1.02 (- 3.31 to 1.26)	- 0.13 (- 3.01 to 2.74)	PD% = - 0.1644 x time (day) r = 0.06 P = 0.614
**- 20 °C**	**1st week**	**2nd week**	**3rd week**	**4th week**	**5th week**	**6th week**	**/**	
PD%	0.10 (- 2.77 to 2.96)	- 2.00 (- 4.34 to 0.34)	- 2.26 (- 4.60 to 0.08)	- 2.35 (- 4.57 to -0.13)	- 2.42 (- 4.59 to -0,24)	- 2.25 (- 4.89 to 0.40)	/	PD% = - 0.5190 x time (week) r = -0.22 P = 0.080
**- 80 °C**	**1st week**	**2nd week**	**3rd week**	**4th week**	**5th week**	**6th week**	**/**	
PD%	1.24 (- 1.78 to 4.25)	- 0.41 (- 2.70 to 1.88)	- 0.29 (- 1.96 to 1.38)	- 0.61 (- 2.47 to 1.24)	0.70 (- 2.05 to 3.45)	2.00 (- 0.86 to 4.86)	/	PD% = 0.1316 x time (week) r = 0.08 P = 0.545
PD - percentage deviations. Cl - confidence interval. P < 0.05 was considered statistically significant.								

Throughout the study, the B-KCAL-CONSET Buhlmann fCAL turbo Control Kit (Buhlmann Laboratories AG, Schonenbuch, Switzerland) at two concentration levels was used to determine for presence or absence of the bias between multiple batches of analysis (*19*). Two IQC lots, 4141 and 2915, were utilized. The CV% for the two concentration levels for two IQC lots were 5.01% for level 1 (target value 83 µg/g) and 2.23% for level 2 (target value 271 µg/g) of IQC lot 4141 and 4.69% for level 1 (target value 84 µg/g) and 3.02% for level 2 (target value 282 µg/g) of IQC lot 2915, respectively. The IQC were analyzed minimum three times a week.

Moreover, additional information, in the form of raw data in Excel format, is provided in the supplementary material for this stability research.

## Discussion

To the best of our knowledge, this is the first study that aimed to determine short- and long-term stability of synovial calprotectin according to the recent CRESS recommendations for conducting stability studies. Calprotectin concentrations in synovial fluid samples remained stable for 7 days at 4-8 °C and for 6 weeks at - 20 °C and - 80 °C. The authors conclude only that the concentration of calprotectin agreed at every time point and in one sample the calprotectin varied from the baseline sample by < 20%. Our findings demonstrated that the PD% for long-term preservation at - 80 °C was less than 10%. Two other studies mentioned storing the synovial fluid samples at a temperature of - 80 °C without specifying the duration of storage, and calprotectin concentration was measured using the ELISA technique in these two experiments (*3*, *4*). This demonstrates the importance of obtaining information on long-term stability at - 20 or - 80 °C, especially because the freezers for - 20 °C storage are more common and used in day-to-day laboratory practice and storage. Alkadhem *et al*. investigated the stability of synovial calprotectin at room temperature and at 4 °C, and it was shown that it was stable for 7 days at both storage temperatures which is in accordance with findings in this study (*11*). Furthermore, none of the mentioned studies were conducted according to CRESS guidelines which is important for standardization of reporting results from all stability studies.

The knowledge of short- and long-term stabilities is crucial regardless of the used methods whether it was used immunoturbidimetry on an automatic analyzer where analysis was usually done within a couple of hours, or ELISA method, where it is usual to collect a specific number of samples and the analysis is performed in batches within few weeks or months. The role of laboratory medicine in healthcare systems is to provide precise, accurate, and trustworthy laboratory findings, despite numerous troubleshooting issues that arise in everyday practice. The laboratory may compel to store patient samples for a certain period in the refrigerator or at lower temperatures (- 20 °C or - 80 °C) for various reasons. Additionally, body fluid samples (such as synovial fluid) are extremely challenging, or perhaps impossible, to redraw in routine laboratory practice. Consequently, the laboratory must have knowledge of the stability of analytes in these samples.

Regarding the study’s limitations, additional synovial fluid samples should have been used to have a broader range of calprotectin concentrations. Moreover, the IQCs were not analyzed continuously all days during the study, and the used reagent was only once calibrated while the stability study was in progress. Afterwards the calibration, IQCs were analyzed in two concentration levels, and it was within the predefined range. The performed calibration could have a minor influence on the obtained results. Also, there is currently no immunoturbidimetric assay for automated analyzer which is available on the market for analyzing the concentration of calprotectin in synovial fluid. The only available assay is intended for measuring calprotectin in fecal and serum samples. Another limitation of this study was not using a reagent designed for analyzing calprotectin in serum samples because the consistency of synovial fluid is more similar to serum than stool sample. Further testing of specific characteristics of the reagent used to measure calprotectin, such as the precision of the method, is also necessary. Furthermore, a detailed description of the included patients in this study was not provided because it was used only as leftover synovial fluid samples and other data from patients were not collected.

In conclusion, the results of calprotectin concentrations in synovial fluid should be reliable if the measurement was done from the samples that were stored within 6 weeks in the freezer or 7 days in the refrigerator.

## Data Availability

The data generated and analyzed in the presented study are available from the corresponding author on request.
